# BioFlow-Insight: facilitating reuse of Nextflow workflows with structure reconstruction and visualization

**DOI:** 10.1093/nargab/lqae092

**Published:** 2024-08-06

**Authors:** George Marchment, Bryan Brancotte, Marie Schmit, Frédéric Lemoine, Sarah Cohen-Boulakia

**Affiliations:** Université Paris-Saclay, CNRS, Laboratoire Interdisciplinaire des Sciences du Numérique, 91405, Orsay, France; Institut Pasteur, Université Paris Cité, Bioinformatics and Biostatistics Hub, Paris, France; Institut Pasteur, Université Paris Cité, Bioinformatics and Biostatistics Hub, Paris, France; Institut Pasteur, Université Paris Cité, Bioinformatics and Biostatistics Hub, Paris, France; Institut Pasteur, Université Paris Cité, CNR Virus Des Infections Respiratoires, Paris, France; Université Paris-Saclay, CNRS, Laboratoire Interdisciplinaire des Sciences du Numérique, 91405, Orsay, France

## Abstract

Bioinformatics workflows are increasingly used for sharing analyses, serving as a cornerstone for enhancing the reproducibility and shareability of bioinformatics analyses. In particular, Nextflow is a commonly used workflow system, permitting the creation of large workflows while offering substantial flexibility. An increasing number of Nextflow workflows are being shared on repositories such as GitHub. However, this tremendous opportunity to reuse existing code remains largely underutilized. In cause, the increasing complexity of workflows constitute a major obstacle to code reuse. Consequently, there is a rising need for tools that can help bioinformaticians extract valuable information from their own and others’ workflows. To facilitate workflow inspection and reuse, we developed BioFlow-Insight to automatically analyze the code of Nextflow workflows and generate useful information, particularly in the form of visual graphs depicting the workflow’s structure and representing its individual analysis steps. BioFlow-Insight is an open-source tool, available as both a command-line interface and a web service. It is accessible at https://pypi.org/project/bioflow-insight/ and https://bioflow-insight.pasteur.cloud/.

## Introduction

Bioinformatics analyses conducted using scripts (e.g., bash or perl) are often challenging to share and execute on different computing environments. The introduction of workflow management systems like Taverna (1) in the early 2000s paved the way for more accessible and shareable workflows. Workflow systems such as Galaxy (2), Snakemake (3) and Nextflow (4) have reshaped the development and execution of bioinformatics analyses. Additionally, container technologies like Docker (5) and Singularity (6) have provided greater control over the software environment, further advancing reproducibility efforts. Bioinformatics workflows have become essential for improving the reproducibility and shareability of bioinformatics analyses (7–9).

In code-based systems such as Nextflow, workflow codes are typically accessible on platforms like GitHub. Furthermore, the nf-core initiative (10,11) provides a set of high-quality workflows. With respect to shareability, repositories designed to gather public workflows have been established. The pioneer platform myExperiment (12) has been succeeded by WorkflowHub (13), collecting a wide array of workflows (from Snakemake, Nextflow, Galaxy, CWL (14) and others). In WorkflowHub, workflows are packaged, registered and exchanged as workflow-centric Research Objects using the RO-Crate specification (15), a standard for aggregating and describing research data, including workflows and associated metadata. Such platforms enable workflow retrieval, allowing the search for already available ones.

The growing availability of high-quality, intricate workflow codes shared online is increasing the average level of reuse of bioinformatics analyses. However, such reuse is currently restricted to *rerun*, where workflows are re-executed as-is, as a whole, without any changes. To build upon already existing codes, broader types of reuse are becoming highly desirable. In *reuse-in-part*, one may want to extract a part of a workflow (e.g., a subworkflow made of one or several steps connected together), treat it as a black box, and integrate it into a new workflow. In *repurpose*, the extracted (sub)workflow may also be modified and adapted before being integrated in a new workflow (8). Such broader types of reuse implies high confidence in the original workflow, which necessitates its thorough comprehension. Understanding *the workflow structure*—the workflow steps and how they are interconnected—is key. Given the code complexity, making such a structure easily accessible remains highly challenging.

The steps of the workflow (and their functionalities) are generally described through documentation. Widespread standardisation of documentation has the potential to improve workflow reuse (8), as illustrated by nf-core workflows adoption of a template (11). However, from one workflow to another, documentations are generally written by different developers, and may thus be heterogeneous in various respects (e.g., level of details, simplification choices). Furthermore, documentation requires frequent updates to mirror the changes made in the sources: when outdated, the documentation does not refer to the current state of the workflow, which may be misleading for the reuser.

As a consequence, extracting and analysing workflows’ structures in a consistent and automated manner is of paramount importance: it offers a standardized representation of workflows in their current implementations, thereby promoting transparency, understandability, and trust, ultimately facilitating their reuse. Interestingly, the need to provide workflow structure has recently been underlined by the ‘Workflows Community Summit 2022’ (16).

Regarding structure extraction in current frameworks, while Taverna and Galaxy (1,2) allow users to inherently visualize the analysis steps of a workflow, Nextflow and Snakemake do not naturally provide such a feature. Alternatively, they provide executed parts of the structure post-execution, thanks to the *execution graph* where nodes represent task execution and edges represent the flow of data between these executions. However, it is important to note that these graphs only represent one execution state of the workflow. Obtaining a workflow’s *specification graph*, in which all of the steps and their interactions are represented, thus permitting a user to get a global view of the workflow, would require unifying all possible execution states. Additionally, each execution graph can only be accessed after a workflow run, meaning that the correct configuration and inputs must be provided to the workflow, thus relying on the user’s prior knowledge and understanding of it.

In this article we introduce **BioFlow-Insight**, a tool to enhance reuse that inspects the code of Nextflow workflows and provides their structure. We focus on Nextflow workflows, due to the well-structured Nextflow community, built notably around the nf-core initiative. **BioFlow-Insight** proceeds to: i) analyze the workflow code, identify its structure (all the steps and their interconnections) and build several graphs at different simplification levels, ii) detect several kinds of errors in the code when present, and iii) generate a RO-Crate (JSON-LD) file describing the workflow and its metadata, for the sake of shareability, all without executing the workflow.

To validate its performance, we applied **BioFlow-Insight** to a corpus of 677 open Nextflow workflows extracted from GitHub, enabling us to check their syntax and structure.

The remainder of the article is organised as follows. The first section describes the **BioFlow-Insight**’s functionalities and implementation. In the second section, we present **BioFlow-Insight**’s outputs, a use case, as well as an analysis on a workflow corpus. In the last section we discuss the results and the usage of **BioFlow-Insight**. Access to all datasets, code, and results mentioned in this paper can be found in the ‘BioFlow-Insight and code availability’ section.

## Materials and methods

### BioFlow-Insight’s functionalities

**BioFlow-Insight** combines three main functionalities, namely, workflow structure reconstruction, workflow errors detection, and RO-Crate generation.

#### Workflow structure reconstruction

Nextflow is a code-based framework allowing the development of scalable, flexible, and reproducible workflows (4). Nextflow offers two language versions, DSL1 and DSL2, the latter being more generic and modular. Despite DSL1’s current lack of support from Nextflow, it remains widely used in GitHub workflows (17). **BioFlow-Insight** thus supports both DSL versions.

Nextflow workflows define two objects: *processes* that constitute the main data analysis tasks (encapsulating specific bioinformatics tasks using calls to scripts, bash commands, tools, etc.) and *operators* that form Nextflow specific data manipulation methods (filtering, grouping, forking, etc.). Processes and operators are interconnected through *channels*, which serve as conduits for data transmission (and oriented according to the data flow).

**Figure 1.**
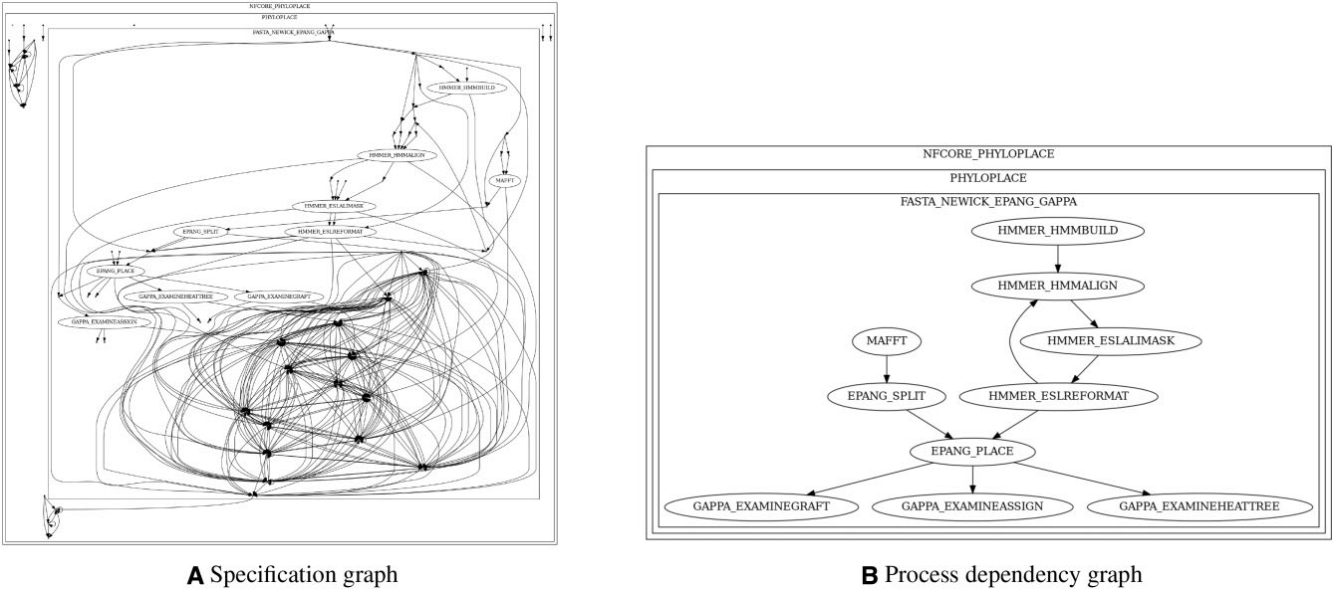
Graph structures generated by **BioFlow-Insight** and representing the phyloplace nf-core workflow. (**A**) The specification graph shows the processes and operations alongside their interactions (without labels). (**B**) The process dependency graph represents the processes and their dependencies. Both graphs are represented without the ‘custom_dumpsoftwareversions’ and the ‘mutiqc’ processes.

**BioFlow-Insight**’s primary functionality is to easily generate the *specification graph* from the workflow’s source code, without the need to run the workflow. In this graph, nodes are either processes or *operations* which are groups of connected operators. We consider two kinds of operations. The *starting operations* are defined as operations without any inputs, they provide (input) data to the workflow. The *following operations* are the remaining operations, with at least one input. Edges in the specification graph represent the relationships between nodes as defined by channels. It is worth noting that the specification graph is not necessarily acyclic (unlike the execution graph). Cycles can form (e.g., see Figure 1) notably since all interactions are depicted and thus non-overlapping (conditional) instances are represented together.

A full representation of the specification graph can be challenging to read and comprehend due to its size and complexity (see Figure 1A), especially for large workflows, with many processes and operations. To address this, **BioFlow-Insight** offers users simplified workflow representations on two levels: i) the *dependency graph*,and ii) the *process dependency graph*. The dependency graph is derived from the specification graph from which following operations are removed, and two remaining nodes are connected if a path exists between them in the original specification graph. The process dependency graph exclusively depicts the processes and their inter-dependencies. This graph is derived from the specification graph by removing all operations, and connecting two nodes (processes) if there exists a path between them in the original specification graph.

Each of the graphs is provided in two text formats (Mermaid (https://github.com/mermaid-js/mermaid) and Dot (https://graphviz.org/doc/info/lang.html) formats), and one image format (PNG) if required.

Finally, **BioFlow-Insight** analyses the generated graphs, and computes various metrics, such as the number of nodes, paths, and the length of the longest path (the full list is available on its website). These metrics are stored as JSON files.

#### Workflow error detection

To generate a workflow’s specification graph, **Bioflow-Insight** must examine all of its code. By doing so, **BioFlow-Insight** can detect programming errors that affect the structure/syntax of the workflow, such as a wrong number of parameters given in a call, a missing file, or a missing element in the code (see Table 1 for the list of detected errors). These types of errors are not necessarily caught by Nextflow during execution, especially if the code involved is not executed (e.g., in a specific configuration state).

**Table 1.** Description of the errors detected by BioFlow-Insight, with the number of workflows that produce the error within the corpus of 677 open public workflows

Errors due to BioFlow-Insight’s limited Scope	
Error description	Number of workflows
Unknown element used in a pipe operator	1
Ternary conditional operator used with a tuple	2
Tuple associated with a call	5
**Errors or ambiguities detected in the workflow’s code**	
**Error description**	**Number of workflows**
Incorrect number of parameters given for a process or a subworkflow call	16
Channel trying to be created with a name already given to an existing element	27
Multiple channels are given by an emit even though only expecting one	1
Tried to access an emit even though the element its emitting has not been called	16
Tried to include a file which does not exist	14
An include was present in a main or subworkflow	1
In a pipe operator, the first element called does not exist	11
Syntax error in the code (e.g., not the same number of opening and closing parentheses)	34
Element (process or subworkflow) is expected to be defined in a file but is not	8
A subworkflow either emits nothing or too many values for use in an operation	20
A subworkflow or process was defined badly (e.g., multiple input sections, multiple main sections...)	10

#### RO-Crate generation

**BioFlow-Insight** generates a RO-Crate (15) JSON-LD file describing the workflow’s metadata and constituent elements. Standardizing the annotation and description of workflows would significantly enhance their sharing, comparison and interrogation; we achieve this by utilizing RO-Crate. Of note, the framework provided by RO-Crate to describe a workflow does not fully accommodate Nextflow workflows in its current state. Therefore, the file generated by **BioFlow-Insight** extends the current specification provided by RO-Crate (see (18)).

### Implementation

**BioFlow-Insight** is an open source tool, implemented in Python 3 with an object-oriented programming approach, and is available as a common-line interface as well as a web interface.

### General pipeline

**Figure 2.**
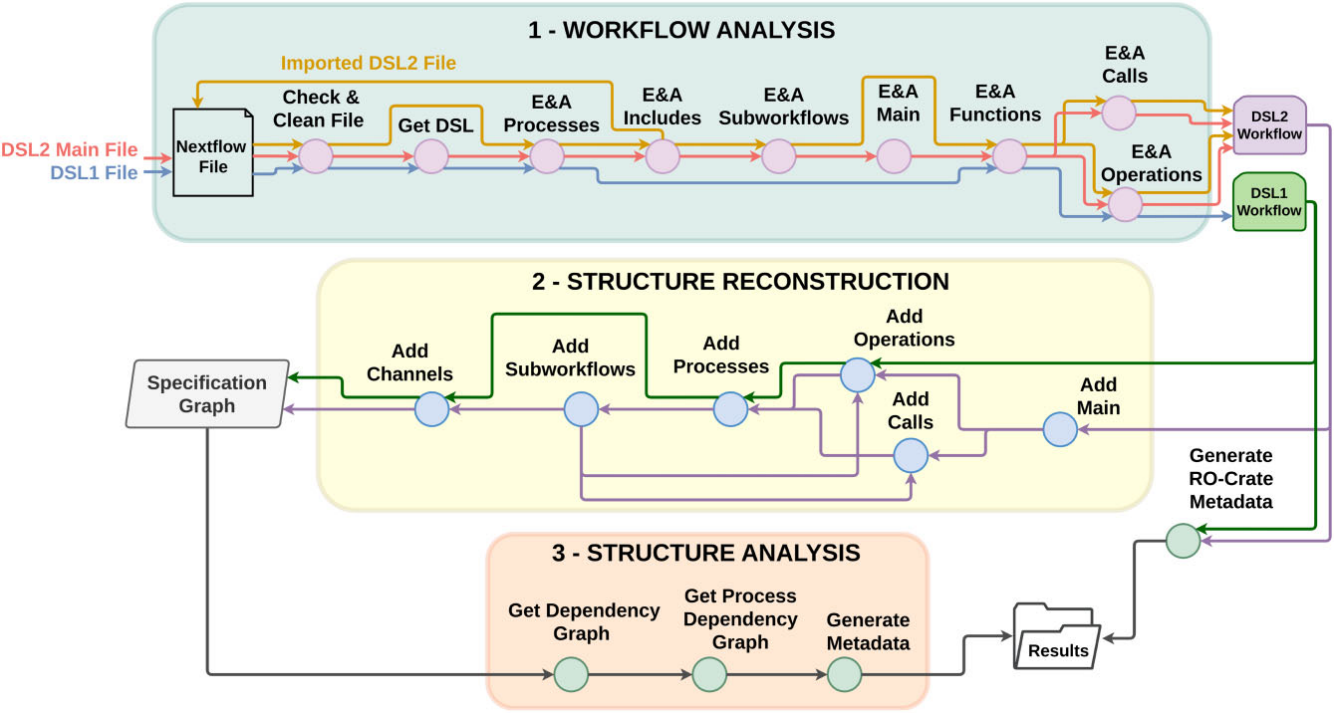
Simplified representation of BioFlow-Insight’s pipeline, which comprises three phases: (1) ‘Workflow Analysis’ parses and analyses the workflow code. Initially, the main Nextflow file provided as input undergoes checks and cleaning procedures, such as removing comments or tallying opening and closing curly brackets. The version of the DSL is then identified (1 or 2), which determines which procedure will follow. For workflows in DSL1 (blue path), processes, functions, and operations are extracted and analysed (E&A) from their unique Nextflow file. For DSL2 workflows, the procedure is slightly more intricate. First, processes are extracted from the main file, and the analysis reruns (yellow path) for each imported file, bypassing the ‘Get DSL’ step. Subworkflows, functions, calls, and operations are all extracted and analysed for each DSL2 file type (red and yellow). Then, the (sub)workflow defined in the main file (red path) is extracted. At the end of this phase, the RO-Crate JSON-LD file is also generated. (2) ‘Structure Reconstruction’ builds the specification graph using all the elements extracted from the workflow’s code. (3) ‘Structure Analysis’ generates the simplified representations of the workflow and also analyses the graphs to obtain their corresponding metadata.

For a given workflow, **BioFlow-Insight** begins by processing the main Nextflow file, typically located at the root of the workflow project. In DSL1 workflows, it analyses the entire unique file to extract processes and operations. In DSL2, the analysis extends to every Nextflow file imported in the project, examining calls to subworkflows and processes, as these elements determine the components used during execution. Figure 2 presents a simplified representation of **BioFlow-Insight**’s pipeline, as well of a complete description of its steps.

## Results

**BioFlow-Insight** analyses the code of a Nextflow workflow without executing it. It then provides assistance to the workflow developers and proceeds to generate several types of outputs.

The first type of output consists of the workflow structure, depicted through graph representations at three levels of granularity. The *specification graph* provides a comprehensive view of the workflow, capturing every element—including all processes, operations, and channels. For large workflows, this graph can become densely packed and challenging to interpret. Two additional graphs gradually simplify this possibly complex representation. Figures 1A and B respectively provide examples of the full and simplified structure of the same workflow.

The second type of output comprises the metrics computed for each of the graphs. These metrics serve primarily for in-depth graph analyses, and assist in further characterising the workflows. For instance, this enables conducting meta-analyses on public workflows, such as examining their size or shape (as indicated by the number of edges, or node degree).

Finally, the last type of output is the RO-Crate JSON-LD file generated by **BioFlow-Insight** describing the workflow and its metadata (such as the keywords, description and authors when provided). Utilising the RO-Crate standard to describe and annotate workflows further increases workflow shareability and, ultimately, reusability. For example, this functionality would streamline the upload of workflows to repositories such as WorkflowHub, which supports the RO-Crate standard.

Even though **BioFlow-Insight** does not provide any output for the *error detection* functionality, it is worth recalling that if there is a programming error in the code that could impact the structure, **BioFlow-Insight** is capable of detecting it. It stops the analysis and provides a detailed description of the error, including a clear message and, where possible, the position in the code, making it easy to correct the workflow. Hence, **BioFlow-Insight** can be used as a Nextflow companion tool to help developers gain a global view of their workflow and pinpoint potential coding errors.

In the following, we present a use case, as well as the results of an analysis on a workflow corpus, both utilizing **BioFlow-Insight**

### Phyloplace: workflow case study

Thanks to the graphs generated by **BioFlow-Insight**, understanding the individual steps of a workflow and how they are connected becomes significantly easier. To illustrate this with an example, let us consider the nf-core’s phyloplace workflow (https://nf-co.re/phyloplace). Phyloplace specializes in phylogenetic placement, i.e., inferring evolutionary relationships of sequences (DNA or protein), within the context of a larger, already existing, phylogenetic tree. With 860 lines of code distributed across 15 different files, efforts to extract and reuse sections of the code could demand significant work to first thoroughly comprehend the code’s structure and the interconnections between its various steps. The full structure of this workflow (corresponding to the specification graph) is shown in Figure 1A. Comprehending it globally is nearly impossible due to its size and complexity (even without showing the labels), a direct consequence of the workflow’s size. However, building the process dependency graph with **BioFlow-Insight** makes the individual steps distinguishable and the workflow global structure understandable. It is even clearer after removing ‘custom_dumpsoftwareversions’ and ‘multiqc’, two processes used for result gathering and software version tracking (see Figure 1B).

On top of the quality of the nf-core workflows implementations, they are also well annotated, making the use of the process dependency graph to examine the individual steps possibly less useful. However, many of the publicly shared workflows lack comprehensive annotations. For these workflows, the use of either the specification graph (for less complex workflows) or one of the simplified graphs can facilitate comprehending their structure, and therefore their functionality, hence facilitating their reusability.

A final point to mention regarding **BioFlow-Insight**’s usefulness is that it allows developers to compare the graphs generated by **BioFlow-Insight**, which are derived from the workflow’s implementation, with the workflow structure drawings in the documentation (often designed during the pre-development phase). This comparison helps developers verify the correctness of the implementation against the original specification and possibly keep the documentation up-to-date, that is, in accordance with any changes in the code.

### Analysis of a large dataset of workflows

To test **BioFlow-Insight**’s validity on a large scale, in the same spirit of previous work (8), we constructed a dataset of 677 publicly available open workflows. This dataset was created using a crawler, which browses GitHub to extract Nextflow workflows with an open license. **BioFlow-Insight** was tested on each of these workflows. The structure was successfully constructed for 75.5% of them. From these workflows, over a hundred were further manually checked to validate the analysis and structures generated.

Of the remaining 24.5% that **BioFlow-Insight** analysed but was unable to generate the graphs, only 8 cases out of the 166 were due to particularly complex workflow code patterns that **BioFlow-Insight** does not support yet. The remaining 158 were due to confirmed code errors or ambiguities in the code (e.g., identically named processes and channels). Table 1 describes the various errors detected by **BioFlow-Insight** along with the number of workflows that produce each error.

## Discussion

In this work, we present **BioFlow-Insight**, a freely accessible bioinformatics tool available both as a common-line interface and a web service. It facilitates Nextflow code-based workflows reuse. The originality of **BioFlow-Insight** lies in its ability to consider a wide range of workflow reuse scenarios, including not only *rerun*, which re-executes the identical workflow without changes, but also *reuse-in-part*, which extracts and reuses specific components and *repurposing*, which involves modifying and adapting the workflow to meet new requirements. To support these scenarios, **BioFlow-Insight** reconstructs the workflow structure at several levels of granularity, offering users a clear understanding of the various workflow steps and their interactions. In addition, it allows developers to easily detect specific types of errors in their code without having to execute it. A last functionality to mention is the ability of **BioFlow-Insight** to generate a shareable RO-Crate representation of the workflow. **BioFlow-Insight** was tested on a large corpus of publicly available Nextflow workflows, where it successfully analysed the majority of them. For the workflows that were not successfully analysed, **BioFlow-Insight** was capable of detecting and explaining the errors, facilitating correction.

With the growing number of available workflows, we are confident that **BioFlow-Insight** will become a valuable resource to address the increasingly important need for workflow reuse.

In future work, we plan to utilise the graphs generated by **BioFlow-Insight** to study the similarities among workflows. Additionally, we believe the structures generated could be useful in describing Nextflow workflows in the Common Workflow Language (14). Finally, we hope to ultimately integrate **BioFlow-Insight** with platforms such as WorkflowHub (13) to facilitate the upload of workflows onto the platform.

## Data Availability

**BioFlow-Insight** source code is freely available at https://gitlab.liris.cnrs.fr/sharefair/bioflow-insight, easily installable via pip as a CLI here https://pypi.org/project/bioflow-insight/ and is callable from its web interface at https://bioflow-insight.pasteur.cloud/. The datasets and the code used to generate the results are available in the following repositories: i) BioFlow-Insight v1.0 https://doi.org/10.6084/m9.figshare.25416100.v1, ii) Workflow Corpus https://doi.org/10.5281/zenodo.10817605, iii) GitHub Crawler https://doi.org/10.6084/m9.figshare.25416109.v1, iv) Phyloplace Workflow Case Study https://doi.org/10.6084/m9.figshare.25416055.v1, v) Analysis of a large dataset of workflows https://doi.org/10.6084/m9.figshare.25416115.v1.
